# Factors associated with successful smoking cessation in men with or without cardiovascular disease or cancer: Nationwide Korean population analysis

**DOI:** 10.18332/tid/159169

**Published:** 2023-02-20

**Authors:** Youngmee Kim, Won-Kyung Cho

**Affiliations:** 1 Red Cross College of Nursing, Chung-Ang University, Seoul, Republic of Korea; 2 International Healthcare Center, Asan Medical Center, University of Ulsan College of Medicine, Ulsan, Republic of Korea; 3 Department of Pulmonary and Critical Care Medicine, Asan Medical Center, University of Ulsan College of Medicine, Ulsan, Republic of Korea

**Keywords:** cancer, cardiovascular disease (CVD), Koreans, factors, smoking cessation

## Abstract

**INTRODUCTION:**

This study was conducted to explore factors associated with smoking cessation in male smokers with cardiovascular disease (CVD) or cancer, the two leading causes of death worldwide, and to compare them with quitting factors in smokers without the two diseases.

**METHODS:**

This is a secondary dataset analysis of the Korea National Health and Nutrition Examination Survey (KNHANES), nationally representative data from 2008–2019 (excluding 2013–2014), and included 12998 men without CVD or cancer (group without CVD or cancer), 1027 men with CVD (CVD group), and 616 men with cancer (cancer group). A Wald test with multiple logistic regression analysis was conducted.

**RESULTS:**

The quitting success rates in the CVD and cancer groups were consistently higher than those in the group without CVD or cancer. Old age and willpower in the CVD group, and old age and being married in the cancer group were associated with quitting success. Secondhand smoking and methods of quitting other than willpower were related to quitting failure in both groups. When interaction effects between the groups were examined, household income was the only factor associated with successful cessation in the group without CVD or cancer (AOR=1.17, 1.18, and 1.40, among the second, third, and fourth highest income quartiles, respectively; p for interaction=0.023). Higher smoking amounts (AOR=0.85; p<0.001) and poor health perception (AOR=0.64; p=0.035) were associated with quitting failure in the group without CVD or cancer. However, no significant factor was detected related to smoking cessation in both the CVD and cancer groups when the interaction effect was investigated.

**CONCLUSIONS:**

The quitting success rates in the CVD and cancer groups were higher, but no disease-specific quitting factors were identified. Therefore, being diagnosed with CVD or cancer itself could be inferred as a motive for quitting smoking.

## INTRODUCTION

Cardiovascular disease (CVD) and cancer are the leading causes of morbidity and mortality worldwide^1^. CVD, including hypertension, coronary artery disease, cerebrovascular disease, and heart failure, accounted for approximately one-third of all deaths in 2015, and is estimated to be associated with nearly 18 million deaths worldwide^2^. Also, it is estimated that 9.6 million people died of various forms of cancer in 2017, making it the second leading cause of death after CVD^3^.

It is well known that tobacco use is the most important preventable cause of CVD^4^ and cancer^5^. Needless to say, smoking cessation is paramount to preventing the development and progression of CVD or cancer. However, quitting smoking is challenging and, unfortunately, many smokers continue smoking even after being diagnosed with CVD or cancer. Therefore, if there are disease-specific factors affecting quitting smoking, identifying these factors may be the first step in helping smokers with CVD or cancer.

Numerous studies have reported that various factors, such as physiological, behavioral, environmental, psychological, cognitive, and social factors, are involved in the success or failure of smoking cessation in the general population^6,7^. Previous studies have also investigated smoking cessation factors in CVD and cancer populations but with some limitations in, for example, study design, objectives, and population size^8-14^.

This study was conducted to explore the disease-specific factors associated with smoking cessation in adult male smokers with CVD or cancer, the two leading causes of death worldwide. To address the study objectives, the Korea National Health and Nutrition Examination Survey (KNHANES), nationally representative data, were analysed^15^. We first investigated smoking cessation-related factors in adult male smokers with CVD, cancer, or neither of the two. We then compared these factors between groups to probe potential interaction effects.

## METHODS

### Study design

This study analyzed data from the Korea National Health and Nutrition Examination Survey (KNHANES), from 2008–2019 (except 2013–2014 due to incomplete data). KNHANES, is an ongoing, nationwide, annual and population-based survey. It is a cross-sectional, multistage, stratified, and clustered probability sampling survey based on geographical region, age, and sex, which investigates the health, lifestyle, and eating habits of Koreans^15^. The survey consisted of three components: health interviews, health check-ups, and nutrition survey. The health interviews and check-ups were conducted by trained medical staff and interviewers at the mobile examination center. A week after the health interview and check-ups, nutritionists paid a visit to the participants’ homes for the nutrition survey. In addition, blood samples were collected at the mobile examination center in the morning after fasting for at least 8 hours.

### Ethics considerations

The data were downloaded through a predetermined registration procedure. Prior to the survey, written informed consent was obtained from each study participant. The study was conducted in accordance with the guidelines of the Declaration of Helsinki. The institutional review board (IRB) of the Korea Disease Control and Prevention Agency (KDCA) reviewed and approved the KNHANES survey. The IRB approval numbers were 2008-04EXP-01-C, 2009-01CON-03-2C, 2010-02CON-21-C, 2011-02CON-06-C, 2012-01EXP-01-2C, 2018-01-03-P-A, and 2018-01-03-C-A.

### Participants

Men (aged ≥19 years) who have smoked more than 100 cigarettes in their lifetime and have tried to quit smoking in the past were included in the study. Among the 85570 people screened, 14641 people satisfying the study criteria were included in the study. The final study population consisted of 12998 men without CVD or cancer (hereafter referred to as ‘group without CVD or cancer’), 1027 men with CVD (hereafter, ‘CVD group’), and 616 men with cancer (hereafter, ‘cancer group’). People with both CVD and cancer were excluded. The criteria for people with CVD or cancer were based on diagnosis by a medical doctor.

**Figure 1:**
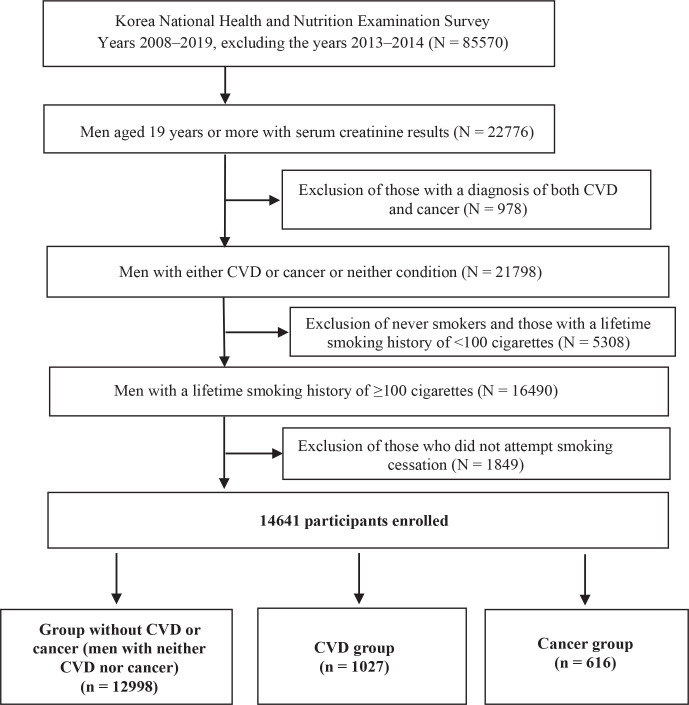
Flow diagram depicting participant selection and the number of participants in the Korea National Health and Nutrition Examination Survey (KNHANES) database

Previous studies reported significant differences between self-reported smoking rates and those assessed by measuring of cotinine levels in Korean women, which might be due to social stigma and prejudice they face^16-18^. Smoking history in the KNHANES was determined relying on self-reported data; thus, this study included only men. Figure 1 demonstrates the flow diagram of the selection process and the number of study participants.

### Definition and measurement of important variables

Several context-specific terms and definitions, largely in accordance with the CDC guidelines, were used in this study^19,20^. We used the term ‘successful quitters’ to refer to the participants who smoked at least 100 cigarettes to date but do not smoke any more (at the time of participation). The term ‘unsuccessful quitters’ describes the participants meeting the following three criteria at the time of participation: 1) the participant smoked at least 100 cigarettes to date; 2) the participant had tried to quit smoking at least once; and 3) the participant still smokes (tried quitting but failed to do so). In addition to current smoking status, additional smoking-related histories were obtained through the questionnaire, such as lifetime smoking amount (pack-years), exposure to secondhand smoke, and smoking cessation methods.

The survey included questions on exercise habits, alcohol consumption, health status, and perceived stress levels. To help the participants provide objective answers, the survey guidelines included examples and well-defined categories. Some questions, on the other hand, relied on their subjective perceptions. For example, ‘regular exercise’ was defined as exercising three times a week, each time for 20 minutes with high intensity or, alternatively, five times a week, each time for 30 minutes with moderate intensity. The definitions of ‘high’ and ‘moderate’ exercise intensities, however, were subjective; an exercise routine that is perceived as hard by the participant because it cannot be completed without getting physically exhausted was considered a ‘high intensity’ exercise. Swimming, heavy weightlifting, and running were considered vigorous exercise, while table tennis was listed as an example of moderate exercise.

Heavy drinkers were defined as those who consume seven or more drinks (regardless of alcohol type) per occasion at least twice a week. Perceived health (multiple levels ranging from very poor to very good), perceived psychological stress (moderate to severe subjective stress), and other parameters were defined as described previously^15,21^. Trauma history was defined as a history of at least one accident or intoxication that required hospitalization and/or emergency room treatment over the past year. Health behaviors refer to alcohol consumption frequency, exercise habits, and eating patterns. Quality of life (QoL) was measured using the EQ-5D system developed by EuroQoL. The EQ-5D index is a measure of health status comprising five dimensions: mobility, selfcare, usual activities, pain/discomfort, and anxiety/depression. The closer the score is to 1, the higher the quality of life. In addition, prevalence of anxiety and depression was also investigated^19^.

### Statistical analysis

All data analyses were performed using SAS version 9.4 (SAS Institute, Inc., Cary, NC, USA), and the data were presented as mean ± standard error (SE) for continuous variables or as proportions ± SE for categorical variables. The prevalence of successful and unsuccessful quitters according to group was presented as a proportion. T-tests and chi-squared tests were conducted to assess the differences between groups for continuous and categorical variables, respectively.

Variables with p<0.1 in univariate analysis were used in multiple logistic regression analysis. Wald test with multiple logistic regression analyses was conducted to identify factors associated with smoking cessation in each group and the interaction effects between groups. The estimates for each interaction term between groups were obtained with the multiple logistic regression analysis adjusting for the involved variables such as age, marital status, occupation, household income, lifetime smoking amount, secondhand smoking, BMI, and smoking cessation methods. A p<0.05 or 95% confidence interval (CI) that did not span 1.0 was considered to indicate statistically significant differences. Data from the KNHANES were derived using stratified and multistage clustered probability sampling methods to represent the entire Korean population, so population weights were applied to the analyses^15^.

Wald test with multiple logistic regression analysis was conducted using the Korea National Health and Nutrition Examination Survey (KNHANES).

## RESULTS

### Prevalence of successful and unsuccessful quitters

**Figure 2:**
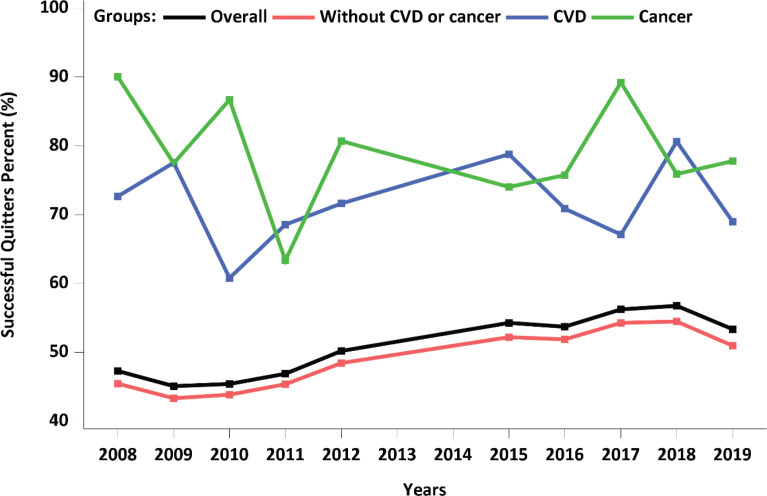
Percentage of successful quitters by subgroups during the study period in the Korea National Health and Nutrition Examination Survey (KNHANES)

Table 1 and Figure 2 show the prevalence of successful and unsuccessful quitters by group. Among a total of 14641 participants, 1027 had CVD and 616 had cancer. Of the remaining 12998 participants without CVD or cancer, 7152 (weighted percentage: 48.9%) were successful in quitting smoking; 812 of 1027 individuals with CVD (weighted percentage: 71.7%) and 509 of 616 individuals with cancer (weighted percentage: 78.7%) were also successful in quitting smoking. Overall, quitting success rates in the CVD and cancer groups were consistently higher than that in the group without CVD or cancer. In addition, the cancer group tended to show a higher rate of success than the CVD group.

**Table 1:** Prevalence of successful and unsuccessful quitters in each group, Korea National Health and Nutrition Examination Survey (KNHANES) (N=14641)

	*Total (N=14641)*			*Group without CVD or cancer (N=12998)*			*CVD (N=1027)*			*Cancer (N=616)*		
*Year*	*Successful quitters (n=8426)^a^*	*Unsuccessful quitters (n=6215)^b^*	*p*	*Successful quitters (n=7152)^c^*	*Unsuccessful quitters (n=5846)^d^*	*p*	*Successful quitters (n=765)^e^*	*Unsuccessful quitters (n=262)^f^*	*p*	*Successful quitters (n=509)^g^*	*Unsuccessful quitters (n=107)^h^*	*p*
2008	47.3 (1.50)	52.7 (1.50)	<0.001	45.5 (1.57)	54.5 (1.57)	<0.001	72.7 (6.08)	27.3 (6.08)	0.299	90.0 (4.09)	10.0 (4.09)	0.193
2009	45.1 (1.34)	54.9 (1.34)		43.3 (1.32)	56.7 (1.32)		77.5 (5.59)	22.5 (5.59)		77.4 (8.25)	22.6 (8.25)	
2010	45.4 (1.70)	54.6 (1.70)		43.8 (1.75)	56.2 (1.75)		60.8 (7.27)	39.2 (7.27)		86.7 (5.43)	13.3 (5.43)	
2011	46.9 (1.67)	53.1 (1.67)		45.4 (1.73)	54.6 (1.73)		68.5 (5.67)	31.5 (5.67)		63.3 (9.97)	36.7 (9.97)	
2012	50.2 (1.62)	49.8 (1.62)		48.4 (1.69)	51.6 (1.69)		71.6 (6.70)	28.4 (6.70)		80.7 (6.27)	19.3 (6.27)	
2015	54.3 (1.52)	45.7 (1.52)		52.2 (1.64)	47.8 (1.64)		78.8 (4.50)	21.2 (4.50)		74.0 (6.82)	26.0 (6.82)	
2016	53.7 (1.86)	46.3 (1.86)		51.9 (1.95)	48.1 (1.95)		70.8 (5.37)	29.2 (5.37)		75.7 (5.95)	24.3 (5.95)	
2017	56.2 (1.66)	43.8 (1.66)		54.3 (1.80)	45.7 (1.80)		67.1 (5.23)	32.9 (5.23)		89.2 (4.10)	10.8 (4.10)	
2018	56.8 (1.42)	43.2 (1.42)		54.5 (1.54)	45.5 (1.54)		80.6 (4.40)	19.4 (4.40)		75.9 (7.38)	24.1 (7.38)	
2019	53.4 (1.63)	46.6 (1.63)		50.9 (1.63)	49.1 (1.63)		69.0 (5.25)	31.0 (5.25)		77.8 (5.54)	22.2 (5.54)	

Data are presented as weighted percentage (standard error, SE). The p-values were determined by the Rao–Scott chi-squared test. Data were not collected in 2013 and 2014. Weighted n (weighted %):

a 6192837 (50.9%);

b 5981988 (49.1%);

c 5493138 (48.9%);

d 5740320 (51.1%);

e 421661 (71.7%);

f 166299 (28.3%);

g 278039 (78.7%); and

h 75369 (21.3%).

### Sociodemographic characteristics and smoking history

Table 2 shows the general characteristics and smoking history of the participants in each group by their smoking cessation status. In all groups, successful quitters were older and more likely to be married compared with the unsuccessful quitters. Lifetime smoking amount was significantly higher among successful quitters in the group without CVD or cancer, but the difference was minimal (p=0.023). Secondhand smoking exposure at home or at work was significantly higher among unsuccessful smokers in both the group without CVD or cancer and the CVD groups. In the cancer group, only secondhand exposure at work was significantly higher in unsuccessful smokers. The smoking cessation method was found to use more willpower among successful smokers in all groups.

**Table 2:** Sociodemographic characteristics and smoking history of participants, Korea National Health and Nutrition Examination Survey (KNHANES) (N=14641)

*Characteristics*	*Group without CVD or cancer (N=12998)*			*CVD (N=1027)*			*Cancer (N=616)*		
	*Successful quitters (n=7152)*	*Unsuccessful quitters (n=5846)*	*p*	*Successful quitters (n=765)*	*Unsuccessful quitters (n=262)*	*p*	*Successful quitters (n=509)*	*Unsuccessful quitters (n=107)*	*p*
**Age** (years)	51.40 ± 0.22	41.57 ± 0.22	<0.001	65.52 ± 0.56	59.86 ± 0.89	<0.001	64.74 ± 0.64	56.81 ± 1.54	<0.001
**Age groups** (years)			<0.001			<0.001			<0.001
19–39	22.0 (0.68)	47.8 (0.84)		2.2 (0.89)	5.5 (1.84)		1.7 (0.88)	6.9 (3.36)	
40–64	57.5 (0.73)	46.5 (0.81)		39.2 (2.20)	58.6 (3.50)		43.3 (2.74)	66.6 (4.99)	
≥65	20.5 (0.53)	5.7 (0.28)		58.6 (2.22)	35.9 (3.38)		54.9 (2.74)	26.5 (4.23)	
**Married**	84.0 (0.57)	64.5 (0.84)	<0.001	88.0 (1.58)	80.8 (2.86)	0.020	90.4 (1.47)	72.9 (5.43)	<0.001
**Education level**			0.964			0.594			0.816
≤High school	60.2 (0.82)	60.1 (0.81)		82.3 (1.74)	80.5 (2.93)		67.9 (2.75)	66.5 (5.56)	
University or higher	39.8 (0.82)	39.9 (0.81)		17.7 (1.74)	19.5 (2.93)		32.1 (2.75)	33.5 (5.56)	
**Occupation**			<0.001			0.033			0.008
Managers/professionals	16.8 (0.61)	17.3 (0.60)		5.2 (0.94)	9.9 (2.43)		8.0 (1.65)	17.2 (4.50)	
Office worker	12.4 (0.49)	12.9 (0.52)		4.1 (0.96)	6.8 (2.06)		6.8 (1.52)	9.4 (3.97)	
Service workers/sellers	11.1 (0.48)	15.1 (0.59)		7.7 (1.28)	5.8 (1.79)		5.5 (1.22)	11.6 (4.21)	
Agriculture/fisheries/labor	37.4 (0.76)	36.4 (0.78)		28.4 (2.00)	33.8 (3.44)		23.0 (2.16)	26.6 (5.05)	
None	22.3 (0.60)	18.4 (0.64)		54.5 (2.26)	43.8 (3.64)		56.7 (2.63)	35.2 (5.29)	
**Rural residence**	17.5 (0.93)	17.1 (1.01)	0.624	20.7 (1.89)	21.7 (3.00)	0.773	23.0 (2.25)	22.6 (4.79)	0.938
**Household income** (quartiles)			<0.001			0.136			0.785
1 Lowest	21.4 (0.61)	27.4 (0.75)		28.2 (2.09)	29.8 (3.27)		24.4 (2.33)	27.8 (5.59)	
2	25.5 (0.65)	25.7 (0.70)		25.2 (1.92)	32.3 (3.48)		23.2 (2.18)	20.7 (4.55)	
3	25.5 (0.62)	24.7 (0.69)		22.7 (1.81)	20.1 (2.84)		25.0 (2.39)	21.0 (4.80)	
4 Highest	27.5 (0.71)	22.2 (0.73)		23.9 (1.89)	17.8 (2.75)		27.4 (2.53)	30.5 (5.08)	
**Lifetime smoking amount** (pack-years)	17.81 ± 0.24	17.10 ± 0.22	0.023	28.24 ± 0.95	26.81 ± 1.11	0.323	28.39 ± 1.12	28.09 ± 2.18	0.900
									
**Secondhand smoking**									
Workplace	30.6 (0.71)	40.2 (0.78)	<0.001	14.7 (1.65)	29.5 (3.63)	<0.001	14.4 (1.89)	28.3 (5.50)	0.005
Home	2.3 (0.23)	7.7 (0.46)	<0.001	1.8 (0.55)	5.9 (1.57)	0.002	3.4 (1.16)	0.8 (0.80)	0.131
**Smoking cessation methods**									
Willpower	93.5 (0.35)	84.4 (0.54)	<0.001	94.8 (1.03)	79.0 (2.94)	<0.001	94.9 (1.18)	85.2 (4.41)	0.003
Nicotine replacement therapy	5.8 (0.34)	16.9 (0.56)	<0.001	4.3 (0.94)	14.8 (2.71)	<0.001	2.7 (0.75)	10.7 (3.92)	0.002
Education/counselling	5.0 (0.31)	11.3 (0.46)	<0.001	4.7 (0.92)	18.2 (2.90)	<0.001	3.0 (0.95)	16.1 (4.36)	<0.001
Smokers’ quitline	0.5 (0.11)	1.1 (0.15)	0.003	0.9 (0.39)	2.3 (1.13)	0.135	0.1 (0.10)	2.7 (1.86)	<0.001

Data are presented as weighted mean ± standard error (SE) or weighted percent (SE). The p-values were obtained by Student’s t-test or Rao–Scott chi-squared. Income quartiles are age- and sex-adjusted.

### Clinical characteristics, health behaviors, perceived health, and quality of life

Table 3 shows the clinical characteristics, health behaviors, perceived health status perceptions, quality of life (QOL) in all groups according to quitting status. In the group without CVD or cancer, almost all variables significantly differed between successful and unsuccessful quitters, even though the differences were minimal. There were fewer number of variables that differed between successful and unsuccessful quitters in the CVD and cancer groups compared with the group without CVD or cancer. In most groups, the many variables related to health behaviors, psychological stress and health-related QoL were significantly worse in failed quitters.

**Table 3:** Clinical characteristics, health behaviors, perceived health, and quality of life of participants, Korea National Health and Nutrition Examination Survey (KNHANES) (N=14641)

*Characteristics*	*Group without CVD or cancer (N=12998)*			*CVD (N=1027)*			*Cancer (N=616)*		
	*Successful quitters (n=7152)*	*Unsuccessful quitters (n=5846)*	*P*	*Successful quitters (n=765)*	*Unsuccessful quitters (n=262)*	*p*	*Successful quitters (n=509)*	*Unsuccessful quitters (n=107)*	*p*
Systolic blood pressure (mmHg)	121.65 ± 0.22	117.78 ± 0.23	<0.001	125.33 ± 0.71	122.70 ± 1.28	0.069	122.11 ± 0.90	122.31 ± 2.25	0.938
Diastolic blood pressure (mmHg)	79.06 ± 0.16	78.11 ± 0.18	<0.001	74.64 ± 0.47	75.41 ± 0.94	0.465	74.15 ± 0.59	76.50 ± 1.31	0.105
Body mass index (kg/m^2^)	24.53 ± 0.04	24.32 ± 0.05	0.002	24.26 ± 0.13	24.15 ± 0.22	0.688	23.33 ± 0.14	23.18 ± 0.35	0.690
Waist circumference (cm)	86.45 ± 0.13	85.20 ± 0.15	<0.001	87.76 ± 0.37	87.27 ± 0.61	0.488	84.99 ± 0.43	84.20 ± 0.95	0.447
Fasting blood sugar (mg/dL)	102.46 ± 0.31	99.49 ± 0.37	<0.001	112.99 ± 1.53	106.50 ± 1.71	0.005	105.95 ± 1.31	101.30 ± 1.81	0.038
Total cholesterol (mg/dL)	192.39 ± 0.53	191.39 ± 0.58	0.203	163.26 ± 1.74	164.08 ± 2.90	0.808	179.09 ± 2.06	189.70 ± 4.66	0.036
LDL cholesterol (mg/dL)	47.54 ± 0.16	46.26 ± 0.17	<0.001	45.25 ± 0.50	42.94 ± 0.89	0.024	46.61 ± 0.64	46.87 ± 1.46	0.871
HDL cholesterol (mg/dL)	116.19 ± 0.47	114.64 ± 0.51	0.025	92.41 ± 1.47	91.22 ± 2.50	0.684	104.54 ± 1.92	111.70 ± 4.14	0.112
Triglyceride (mg/dL)	158.01 ± 1.97	176.39 ± 2.34	<0.001	136.23 ± 3.80	166.97 ± 9.30	0.002	152.44 ± 8.46	174.64 ± 18.38	0.274
Haemoglobin (g/dL)	15.17 ± 0.02	15.55 ± 0.02	<0.001	14.47 ± 0.06	14.79 ± 0.13	0.030	14.35 ± 0.09	14.77 ± 0.19	0.044
Creatinine (mg/dL)	0.98 ± 0.01	0.95 ± 0.00	<0.001	1.06 ± 0.02	0.99 ± 0.02	0.011	1.00 ± 0.01	0.95 ± 0.02	0.021
Hypertension	36.6 (0.66)	23.4 (0.66)	<0.001	63.9 (2.11)	52.0 (3.73)	0.005	42.3 (2.59)	36.7 (5.63)	0.377
Diabetes mellitus	12.4 (0.44)	9.3 (0.43)	<0.001	33.3 (2.12)	27.2 (3.33)	0.128	20.5 (2.09)	14.7 (3.72)	0.196
Dyslipidaemia	17.7 (0.53)	13.0 (0.54)	<0.001	32.7 (2.18)	32.6 (3.73)	0.988	16.3 (2.05)	17.7 (4.34)	0.768
Trauma history	7.1 (0.39)	9.8 (0.46)	<0.001	6.0 (1.04)	4.8 (1.43)	0.512	6.9 (1.33)	5.6 (3.07)	0.705
Heavy drinking	19.9 (0.58)	29.1 (0.71)	<0.001	11.0 (1.46)	15.6 (2.80)	0.109	7.8 (1.41)	24.5 (5.36)	<0.001
Regular exercise	39.5 (0.74)	36.0 (0.77)	<0.001	29.5 (2.01)	23.0 (3.12)	0.094	34.5 (2.52)	49.0 (5.78)	0.016
Sleep duration (h/day)	6.98 ± 0.02	7.02 ± 0.02	0.192	7.01 ± 0.07	6.93 ± 0.12	0.551	7.06 ± 0.09	7.24 ± 0.19	0.377
**Skipping meals**									
Skipping breakfast	16.5 (0.60)	33.2 (0.77)	<0.001	6.5 (1.22)	16.2 (3.09)	<0.001	6.8 (1.49)	25.2 (5.56)	<0.001
Skipping lunch	5.4 (0.33)	9.2 (0.49)	<0.001	4.7 (0.94)	8.7 (2.01)	0.044	6.1 (1.43)	11.5 (3.92)	0.128
Skipping dinner	3.3 (0.26)	4.9 (0.33)	<0.001	2.5 (0.72)	1.9 (0.90)	0.605	3.6 (1.14)	3.9 (2.18)	0.900
**Perceived health status**			<0.001			0.107			0.130
Very good/good	40.6 (0.69)	34.0 (0.75)		17.2 (1.60)	12.2 (2.41)		25.2 (2.38)	34.9 (5.63)	
Fair	46.0 (0.71)	50.5 (0.77)		40.6 (2.23)	37.6 (3.56)		43.2 (2.74)	43.6 (5.93)	
Poor/very poor	13.4 (0.48)	15.5 (0.54)		42.2 (2.14)	50.2 (3.60)		31.6 (2.44)	21.5 (4.90)	
Perceived psychological stress	22.0 (0.61)	32.0 (0.72)	<0.001	17.2 (1.70)	28.1 (3.36)	0.002	16.9 (2.12)	26.5 (5.46)	0.067
EuroQoL: Anxiety/depression	6.2 (0.33)	6.2 (0.36)	0.923	15.4 (1.62)	18.8 (2.79)	0.271	10.0 (1.51)	6.4 (2.75)	0.307
EQ-5D index	0.962 ± 0.001	0.968 ± 0.001	<0.001	0.876 ± 0.008	0.882 ± 0.010	0.663	0.922 ± 0.006	0.952 ± 0.010	0.011

Values presented are weighted mean ± standard error (SE) or weighted percentage (SE). The p-values were determined by Student's t-test or Rao–Scott chi-squared test. Health behaviors refer to alcohol consumption frequency, exercise habits, and eating patterns.

### Factors associated with smoking cessation

Table 4 shows the factors associated with smoking cessation. In the group without CVD or cancer, several factors were associated with successful smoking cessations. To name a few, old age, being married, higher household income, using willpower to quit, higher BMI, regular exercise, and some comorbidities were positively correlated with successful quitting. On the other hand, higher lifetime smoking amount, secondhand smoking, using smoking cessation methods other than willpower, unhealthy behaviors, poor perceived health, and psychological stress were negatively associated with successful smoking cessation.

**Table 4:** Factors associated with smoking cessation in subgroups within the Korea National Health and Nutrition Examination Survey (KNHANES) datasets assessed

*Characteristics*	*Group without CVD or cancer*		*CVD*		*Cancer*		*p for interaction*
	*AOR (95% CI)*	*p*	*AOR (95% CI)*	*p*	*AOR (95% CI)*	*p*	
**Age groups** (years)							0.774
19–39 (Ref.)	1		1		1		
40–64	2.12 (1.87–2.41)	<0.001	3.46 (0.92–13.05)	0.067	2.76 (0.62–12.32)	0.183	
≥65	4.66 (3.84–5.64)	<0.001	6.16 (1.60–23.80)	0.008	6.80 (1.53–30.31)	0.012	
**Marital status** (married)	1.98 (1.74–2.25)	<0.001	1.45 (0.80–2.61)	0.216	2.60 (1.28–5.30)	0.008	0.425
**Occupation**							0.445
Managers/professionals (Ref.)	1		1		1		
Office workers	1.05 (0.89–1.24)	0.576	1.51 (0.47–4.87)	0.486	2.00 (0.50–8.10)	0.329	
Service workers/sellers	0.89 (0.74–1.06)	0.187	1.90 (0.57–6.37)	0.297	1.54 (0.42–5.66)	0.517	
Agriculture/fisheries/labor	1.01 (0.88–1.17)	0.853	1.28 (0.61–2.70)	0.509	2.78 (1.03–7.52)	0.044	
None	1.23 (1.03–1.47)	0.025	1.43 (0.69–2.97)	0.334	3.62 (1.45–9.07)	0.006	
**Household income** (% in quartiles)							**0.023**
1st (lowest) (Ref.)	1		1		1		
2nd	1.17 (1.02–1.35)	**0.025**	0.70 (0.41–1.17)	0.172	1.20 (0.50–2.87)	0.676	
3rd	1.18 (1.03–1.37)	**0.019**	0.96 (0.56–1.65)	0.881	0.95 (0.41–2.21)	0.909	
4th (highest)	1.40 (1.21–1.61)	**<0.001**	1.14 (0.65–2.00)	0.653	0.49 (0.24–1.02)	0.055	
**Lifetime smoking amount** (each 10 pack–year increase)	0.85 (0.83–0.88)	**<0.001**	1.03 (0.94–1.13)	0.538	0.94 (0.83–1.07)	0.357	**<0.001**
**Secondhand smoking**							
Workplace (yes)	0.88 (0.79–0.98)	0.022	0.64 (0.37–1.09)	0.103	0.91 (0.45–1.82)	0.784	0.503
Home (yes)	0.48 (0.36–0.65)	<0.001	0.42 (0.18–0.97)	0.043			0.747
**Smoking cessation methods**							
Willpower	1.51 (1.26–1.80)	<0.001	3.19 (1.70–5.97)	<0.001	1.74 (0.61–4.98)	0.304	0.072
Nicotine replacement therapy	0.45 (0.38–0.54)	<0.001	0.42 (0.20–0.89)	0.023	0.43 (0.16–1.19)	0.104	0.978
Education/counselling	0.56 (0.46–0.68)	<0.001	0.36 (0.18–0.72)	0.004	0.19 (0.06–0.65)	0.008	0.127
Smokers’ quitline	0.81 (0.48–1.38)	0.440	1.67 (0.32–8.60)	0.539	0.04 (0.00–0.71)	0.028	0.086
**Health status**							
BMI (kg/m^2^)	1.06 (1.04–1.08)	<0.001	1.06 (0.99–1.14)	0.102	1.05 (0.95–1.17)	0.328	0.992
Triglyceride (each 10 mg/dL increase)	0.99 (0.99–0.99)	<0.001	0.98 (0.96–1.00)	0.016	1.01 (0.98–1.03)	0.533	0.130
Haemoglobin (g/dL)	0.84 (0.80–0.87)	<0.001	0.93 (0.80–1.09)	0.370	0.92 (0.76–1.11)	0.379	0.296
Creatinine (mg/dL)	1.45 (1.05–2.00)	0.024	1.26 (0.67–2.37)	0.475	2.46 (0.55–11.06)	0.239	0.719
Hypertension	1.32 (1.18–1.47)	<0.001	1.37 (0.91–2.07)	0.126	1.24 (0.70–2.19)	0.466	0.955
Diabetes mellitus	0.89 (0.77–1.04)	0.153	1.27 (0.82–1.95)	0.282	1.13 (0.54–2.38)	0.748	0.293
Dyslipidaemia	1.27 (1.10–1.45)	<0.001	1.16 (0.74–1.81)	0.522	0.85 (0.36–1.97)	0.698	0.619
Trauma history	0.82 (0.69–0.98)	0.027	1.21 (0.56–2.62)	0.633	1.19 (0.31–4.52)	0.797	0.559
Heavy drinking	0.76 (0.67–0.85)	<0.001	0.99 (0.59–1.66)	0.956	0.49 (0.23–1.06)	0.071	0.345
Regular exercise	1.24 (1.13–1.37)	<0.001	1.59 (0.99–2.55)	0.056	0.65 (0.37–1.16)	0.144	0.056
**Skipping meals**							
Skipping breakfast	0.61 (0.54–0.69)	<0.001	0.49 (0.23–1.04)	0.064	0.60 (0.24–1.49)	0.271	0.840
Skipping lunch	0.64 (0.53–0.78)	<0.001	0.86 (0.34–2.23)	0.764	0.73 (0.25–2.14)	0.567	0.815
Skipping dinner	0.80 (0.64–1.01)	0.055	2.67 (0.60–11.93)	0.198	1.19 (0.33–4.33)	0.794	0.253
**Perceived health status**							**0.035**
Very good/good (Ref.)	1		1		1		
Fair	0.76 (0.68–0.84)	**<0.001**	0.90 (0.50–1.63)	0.740	1.22 (0.62–2.40)	0.560	
Poor/very poor	0.64 (0.55–0.75)	**<0.001**	0.60 (0.34–1.08)	0.087	2.08 (0.98–4.39)	0.056	
Perceived psychological stress	0.78 (0.70–0.88)	<0.001	0.78 (0.45–1.35)	0.371	0.73 (0.35–1.49)	0.387	0.980
EuroQoL: anxiety/depression	1.15 (0.92–1.44)	0.220	0.88 (0.51–1.51)	0.637	2.24 (0.61–8.23)	0.226	0.382
EQ–5D index	1.08 (0.56–2.09)	0.817	1.75 (0.48–6.39)	0.395	0.09 (0.00–1.74)	0.110	0.182

The p-values were obtained by the Wald test with logistic regression. All estimates for all interaction terms between groups were obtained from the multiple logistic regression after adjustment for all age, marital status, household income, lifetime smoking amount, secondhand smoking, and smoking cessation methods. Diastolic blood pressure and waist circumference were omitted from the analysis because of multicollinearity. AOR: adjusted odds ratio. Ref: reference.

In both CVD and cancer groups, the number of factors associated with quitting smoking were lower than that in the group without CVD or cancer. Old age and the use of willpower were positively associated with successful quitting in the CVD group, whereas old age, being married, and employment in agriculture, fisheries, or labor were associated with successful smoking cessation in the cancer group. Interestingly, receiving education and counselling for quitting were negatively related to successful smoking cessation in both CVD and cancer groups. Using nicotine replacement therapy and secondhand smoking at home were also negatively associated with quitting in the CVD group. Calling a quitline was related to unsuccessful smoking cessation in the cancer group.

When interaction effects between the three groups were examined, household income was the only factor positively associated with successful smoking cessation in the group without CVD or cancer; smoking cessation rates were 1.17, 1.18, and 1.40 times higher, respectively, in participants in the second, third, and fourth (highest) percentiles of the income quartiles (p for interaction=0.023). On the other hand, higher lifetime total smoking amount was negatively associated with successful quitting in the group without CVD or cancer (AOR=0.85; 95% CI: 0.83–0.88; p for interaction <0.001). Perceived health status was also negatively associated with successful quitting, indicating that fair and poor/very poor health perception, compared with very good/good health perception, was negatively associated with successful smoking cessation in the group without CVD or cancer (AOR of fair perception, 0.76; 95% CI: 0.68–0.84; AOR of poor/very poor perception, 0.64; 95% CI: 0.55–0.75; p for interaction=0.035). No significant factor was detected related to smoking cessation in both the CVD and cancer groups when interaction effect was investigated.

## DISCUSSION

This study investigated and compared factors related to smoking cessation in Korean male smokers with CVD, cancer, or neither. The quitting success rates in the CVD and cancer groups were consistently higher than those in the group without CVD or cancer throughout the study period (Table 1 and Figure 2). When we examined factors associated with the smoking cessation, fewer factors were related to smoking cessation in both the CVD and the cancer groups compared with the group without CVD or cancer. Old age and using willpower to quit in the CVD group, and old age, being married, and employment in agriculture, fisheries, or labor in the cancer group, were the only factors associated with quitting success. Interestingly, receiving education and counselling on quitting were negatively correlated with quitting success in both the CVD and the cancer groups. Using nicotine replacement therapy and secondhand smoke exposure in the CVD group, and calling a quitline in the cancer group were related to failure in quitting. In short, old age, being married, and the use of willpower were related to successful smoking cessation in the CVD and/or cancer groups. Methods of quitting other than willpower were associated with unsuccessful attempts of smoking cessation in either the CVD and/or cancer groups. We do not have a good explanation for this observation. The bottom line is that various smoking cessation methods, other than willpower in the CVD group, did not help smokers in the CVD and cancer groups quit smoking. This may simply implicate that willpower may be the most effective resource to quit smoking in these groups. Since the above factors were also significant factors in the group without CVD or cancer, there were no CVD-specific or cancer-specific differences other than the number of factors that are related to smoking cessation when compared to the group without CVD or cancer.

We also analyzed the interaction effects between the three groups. In the group without CVD or cancer, household income was the only factor positively associated with successful smoking cessation, and higher lifetime smoking amount and poorly perceived health status were the only factors negatively related to successful quitting. No significant factors were detected related to success or failure in quitting in both the CVD and cancer groups when interaction effects were investigated.

Overall, there seems to be no CVD-specific or cancer-specific factors that influence smoking cessation. However, given that the quitting success rates were higher in both the CVD and cancer groups over the study period, compared with the group without CVD or cancer, it can be speculated that having CVD or cancer was the main reason for quitting in this study population.

Continuous smoking among cancer survivors and patients with CVD is not uncommon although smoking cessation is one of the most important measures to prevent relapse of disease. The prevalence of smoking among cancer survivors has been reported to be between 9% and 33%^20,22-28^. Around 50% of smokers do not quit smoking even after a diagnosis of CVD^12,13,29,30^.

Several studies have examined the factors associated with continuous smoking in patients with cancer or CVD. To name a few, younger age, education level (lower), being uninsured, sex (male)^8^, younger age at cancer diagnosis, low socioeconomic status, heavy smoking, diagnosis of non-smoking related cancer, high serum glucose level^9^, nicotine withdrawal symptoms, pain, fatigue, nausea, depression, and anxiety^10,11^, have been reported as factors associated with continued smoking in cancer patients. A higher smoking amount and longer smoking duration before the diagnosis, were associated with persistent smoking after the diagnosis of CVD^12^. Having a life partner^13^ or participating in a cardiac rehabilitation^14^ were related to successful smoking cessation in the CVD patients. Overall, the factors reported to contribute to smoking cessation appear to vary depending on study settings or populations.

The factors associated with smoking cessation in the group without CVD or cancer were more or less the same as previously reported in the general population^30-32^. Among these significant factors, household income was the only factor positively associated with successful smoking cessation, and higher lifetime smoking amount and poorly perceived health status were the only factors negatively related to successful quitting in the group without CVD or cancer when the interaction effect between groups was investigated. Although these factors have already been reported to influence smoking cessation, it is quite intriguing that only these factors were found to be significant in the group without CVD or cancer when the interaction effect between groups was examined^21,31-33^. Future studies are needed to determine whether house income, lifetime smoking amount, and health status perception become more important to smokers without CVD or cancer for quitting smoking.

### Limitations

This study has some limitations. Factors influencing smoking cessation in cancer survivors are known to be related to the type of tumor, the causal relationship between smoking and the diagnosed cancer, and the nature of cancer treatment^34,35^. It is also possible that the quitting success varies according to the type or treatment of CVD. However, such detailed clinical information was not available for analysis. In addition, the data on when the participants stopped smoking and when they were diagnosed with cancer or CVD were not available. We presumed that being diagnosed with CVD or cancer was the main reason for quitting in this study population. Therefore, the timing of quitting in relation to the timing of CVD or cancer diagnosis, if known, could provide a stronger support for our conclusions.

## CONCLUSIONS

The quitting success rates in the CVD and cancer groups were consistently higher than those in the group without CVD or cancer throughout the study period. However, no disease-specific factors influencing smoking cessation were detected in the participants with CVD or cancer in this study population. Given that smoking cessation success rates were consistently higher in both the CVD and cancer groups than in the group without CVD or cancer, being diagnosed with CVD or cancer itself could be inferred as the motive for quitting smoking. Therefore, it is important for healthcare providers to motivate smokers with CVD or cancer, by emphasizing the importance of quitting smoking.

## Data Availability

The data supporting this research are available from the authors on reasonable request.
